# The use of Messenger for research collaboration: An auto-ethnographic study

**DOI:** 10.3389/fpsyg.2022.1076340

**Published:** 2023-01-10

**Authors:** Dennis Alonzo, Cherry Zin Oo

**Affiliations:** ^1^School of Education, University of New South Wales, Kensington, NSW, Australia; ^2^Department of Educational Psychology, Yangon University of Education, Yangon, Myanmar

**Keywords:** social media, Messenger, academic work, publishing, collaboration

## Abstract

The use of social media for the collaboration of academics has been increasing in recent years. However, there are no reported studies on using Messenger as a collaborative platform to write and publish journal articles and apply for research and development grants. We use an auto-ethnography to reflect on our experiences over the last 3 years, using Messenger as our medium for our ongoing collaborative research activities. Our results highlight the benefits and challenges of using social media for this engagement. The capabilities of Messenger, as opposed to traditional correspondence through email, have paved our preference to use this platform. We can engage in dynamic collaboration and focussed discussion with less formal communication conventions through Messenger. In addition, the extra features, including easy phone calls, sending links, resources and screenshots, and using emojis and stickers for more socially cohesive interactions, are valued features of Messenger. We used the activity theory to highlight the interrelationships of factors (i.e., personal, social-emotional, structural, technological, and organisational) contributing to the success of collaborative academic activities, including the successful publication of journal articles and securing research and development grants. The findings of our study significantly contribute to understanding how social media can be effectively used for academic engagement.

## 1. Introduction

The use of social media in education is increasing as it provides alternative platforms for learning, teaching, and assessment (Miller and Olthouse, 2013; Kio, 2015; Yuk and Yunus, 2021). The preference for using social media to support learning and teaching activities is underpinned by the preferences of teachers and students due to their affordances, including their capacity for both synchronous and asynchronous engagements (Breunig, 2016), easy navigation (Tran and Lyon, 2017) and linking to other online resources (Gorska et al., 2020), accessibility (Onuoha et al., 2021), and interactivity (Kaplan and Haenlein, 2014).

There are also other uses of social media besides learning, teaching, and assessment. Some studies demonstrate the intersections between social media and research and engagement. For example, Jordan (2022) explores academics’ perception of what constitutes research impact through social media and how different platforms mediate their perception. There is also evidence that academics use social media for other purposes besides learning and teaching. These include professional development (McPherson et al., 2015; Dermentzi et al., 2016) for research collaboration, including finding, interacting, and supporting other academics and working collaboratively with other researchers (Jordan and Weller, 2018), enhancement of their reputation (Knight and Kaye, 2016), networking (Dermentzi et al., 2016; Donelan, 2016), and sharing of research output (Elsayed, 2016). Whilst academics’ use of social media continues to rise, the theorisation on how academics use it for research collaboration, particularly collaborative writing to enhance their publication remains lacking.

Collaborative writing is composed of several activities, including brainstorming, conceptualising, outlining, drafting, reviewing, revising, and editing (Berndt, 2011). Collaborative research writing is effective in meeting the academics’ demand for publication. It offers a supportive environment, maintains momentum, enhances individual and team motivation, and increases the accountability of everyone to complete the assigned task (Ness et al., 2014). More traditionally way, these processes occur face-to-face. However, given the demands for international collaborations and researchers’ geographical location, these processes shifted online, allowing for greater flexibility and wider reach for other researchers who want to engage in producing academic papers together. In the digital age, collaborative writing has become accessible, providing people with various alternative platforms, including social media (Suominen and Jussila, 2018).

We aim to reflect on our experiences using Messenger for a specific type of collaboration in our academic work. Specifically, through reflection and analyses of our personal experiences, we provide critical insights into how Messenger has been pivotal in accomplishing our aims to publish journal articles and apply for a research grant. We answer the following research questions:

1. What are our motivations for engaging in collaborative research publication?
2. How does Messenger facilitate accomplishing our goals of publishing papers?

To provide context for this paper, the first author is a senior lecturer at one of the top universities in Australia who has supervised the second author in her Ph.D., a lecturer at one of the universities in Asia. Before using Messenger for our collaboration, we used email and Zoom to collaborate in our previous publications. Our shift to using Messenger is driven by our aim to increase our publications, which we need a more accessible and helpful platform. Our use of Messenger has been influential in our success for 1 year in publishing four research articles and one educational blog and securing research and development funds within 1 year. Most of our communication and interactions happen in Messenger, from conceptualising, writing, finalising, and editing our papers. In this paper, we focus on how Messenger enables and supports the processes, products, engagement, and commitment for ongoing collaborative work to publish and secure research grants.

## 2. Literature review

In this section, we present our literature review on the use of social media for research collaboration, the nature of collaborative writing and the emerging evidence that support the use of social media for collaborative research writing.

### 2.1. Social media for research collaboration

The use of social media in daily activities has increased, and Statista (2021) predicts that social media users will be 4.41 billion worldwide by 2025. Waite (2021) highlights using the Internet as a successful tool for research purposes. In the academic field, although most researchers use social media for everyday activities rather than research and teaching (Gu and Widén-Wulff, 2011), there are reports demonstrating the use of social media for research collaboration.

Social media have been an effective tool for researchers to communicate and collaborate with other researchers (Onuoha et al., 2021), including seeking information and interacting socially (Lamberton and Stephen, 2016). In addition, they are useful for identifying research opportunities and disseminating research findings (Rowlands et al., 2011). Researchers have better access to calls for research grants and research findings shared in social media. Furthermore, the use of social media shapes researchers’ identities (Lamberton and Stephen, 2016). Their profiles become more prominent, and their research programs and outputs are widely circulated and known in the academic community.

The use of social media for research collaboration allows for greater flexibility. They enhance collaboration and sharing of documents with other researchers regardless of location (Hobson and Cook, 2011; Skaržauskienė et al., 2013). Researchers in different time zones can take advantage of an asynchronous collaboration (Breunig, 2016). The virtual collaboration offered by social media enhances researchers’ productivity (Chui et al., 2012). There is also a report that engaging in social media can enhance trust, which is an important foundation for collaboration (Calefato et al., 2013). The openness of each other on social media makes them trust their collaborators.

Examples of social media used for research collaboration include Facebook, Twitter, LinkedIn, WhatsApp, ResearchGate, and Mendeley, have been used. These social media platforms enhance information sharing, informal scholarly communication, and research collaboration (Thelwall and Kousha, 2015). Kuteeva (2016) highlights that “Twitter is used to mediate the daily routines of scientific work and to keep researchers and collaborators connected, often at academic events” (p. 440). Researchers use Facebook to disseminate research findings, including advertising their publications (Kortelainen and Katvala, 2012) and sharing information (Junco, 2012). Onuoha et al. (2021) found that WhatsApp is the most popular social media platform for research communication, networking and sharing documents. Other platforms are also used. According to Gorska et al. (2020), researchers use platforms that specialise in academia, although the interaction activities are not as well-developed as Facebook or Instagram. For example, the use of ResearchGate and Mendeley have been used more by researchers to network with an academic audience.

### 2.2. The nature of collaborative research writing

Collaborative research writing is “an iterative and social progress that involves a team focussed on a common objective that negotiates, coordinates, and communicates during the creation of a common document” (Lowry et al., 2004, p. 72). In other words, collaborative writing is a social activity undertaken by people working on producing a shared document (Gimenez and Thondhlana, 2012). According to the strategies highlighted by Lowry et al. (2004), there are single author writing (only one person writes the paper on behalf of a team), sequential writing (a sequence has written a paper of authors involved in a paper), parallel writing (more than one author work on the paper at the same time), and reactive writing (authors in a paper react and adjust to each other’s writing). In a more systematic way, collaborative writing is initiated when people start brainstorming about the possibility of writing a research paper together and completed when revising, editing, submitting, and responding to the reviewers’ feedback (Berndt, 2011). The whole cycle may take longer, and the collaboration may even continue after the paper has been published.

Collaborative research writing is one of the strategies used to increase academics’ number of journal articles published. Ness et al. (2014) argue that collaborative research writing offers wider benefits than sole authorship. When academics agree to venture into co-authorship, the collaborative nature of writing offers supportive environment for all while increasing the accountability of everyone to complete their assigned tasks on time. Apart from the actual writing processes, there are other processes occurring that impact the success of collaborative research writing. According to Rhodes and Lin (2019), the negotiation of roles and responsibilities is sometimes difficult, and hence, usually, the collaboration is “based on a foundation of friendship and a shared belief in the co-construction of knowledge (p. 72).” They recommend that collaborators build mutual respect and shared values before starting a collaborative research writing work.

### 2.3. Affordances of social media for collaborative research writing

In the study of McGrath (2013), McGrath highlighted the importance of collaborative writing and the effects of digital tools. Similarly, Hynninen (2018) investigated the impact of digital tools on research writing. In Hynninen’s study, the participants used Slack, a team collaboration tool, to organise writing in the research group. Then, they used Overleaf, a cloud-based collaborative writing tool, for collaborative writing. For communication purposes, they used email, Skype, and Twitter in writing, evaluating and discussing the results and achievements. Also, Facebook was used for private communication among participants. Twitter was used for different functions, including finding and sharing research-related resources.

Social media have affordances that can facilitate collaborative research writing. The interactive platforms of social media provide an opportunity for academics to discuss and negotiate their ideas (Bjørn and Ngwenyama, 2009). Social media better support the dynamic nature of discussion and negotiation compared to face-to-face collaborative research writing. Also, the possibility of collaborating with other academics in many parts of the world will provide better access for new academics to experts’ knowledge, skills, and experiences from more senior academics (Gorska et al., 2020). Woolley et al. (2010) argue that emerging researchers have the limited social capital to involve in international teams. Thus, if there is a collaboration between early career researchers and experienced researchers, early career researchers will be guided and taught by more experienced researchers (Waite, 2021). However, some researchers may have competitive pressures in collaboration using social media. As their research identities become more visible online, other collaborators tend to disengage due to competition and comparison (Jamali et al., 2014).

Although different types of social media are evident in literature, to the best of our knowledge, there have been little to no studies about using Messenger as a platform for collaborative research writing.

## 3. Materials and methods

We adopt an autoethnography research design to critically reflect on our experience for the last 2 years in using Messenger for collaboration to write and publish journal articles and apply for research and development grants. Autoethnography is a research method that “entails the scientist or practitioner performing narrative analysis pertaining to himself or herself as intimately related to a particular phenomenon” (McIlveen, 2008, p. 15). This research methodology has been applied in various context including transdisciplinary research in collecting and analysing self-reflection from social and natural scientists for transformative change (Haeffner et al., 2022), examining professional identity tensions of transnational teachers (Yazan et al., 2022), exploring the experiences of international students to transitioning to an academic job in the university (Consoli et al., 2022), bringing perspectives of humanities into computer education (Bernard, 2022), leading a whole-school reform (Alonzo et al., 2021), and many others.

### 3.1. Theoretical framework

Engeström’s (1987) activity theory was chosen as a framework for this study, based on Vygotsky’s (1978) conceptualisation of the primacy of culture rather than individual cognition in mediating action, learning and meaning-making. In this theory, the social interactions of individuals within the community facilitate the activity. This model is useful for understanding how different factors influence various socially and culturally mediated activities to achieve the intended outcomes.

Activity theory has been applied in many areas of social research and human-computer interactions, including implementing educational reforms (Alonzo et al., 2021), sustaining professional learning partnerships (Bloomfield and Nguyen, 2015), describing and analysing out-of-school learning through digital learning (Li et al., 2022), explaining the increasing processes of unbundling, digitisation and marketisation in higher education (Cliff et al., 2022), and among others.

This theory describes the roles of the objects (experiences, knowledge, and physical products), tools (documents, resources, etc.), and community (people or stakeholders). The subjects, the people engaged in the activity, work as part of the community to achieve the activity’s objective or outcome. The quality of the interactions among objects, tools, and the community determine the quality of the outcomes. Thus, this analytical framework is useful for reflecting on different elements of social learning systems to understand the patterns of social activities and development, consequently bringing the intended outcomes.

The activity theory is useful in our auto-ethnographic study. The research collaboration we have undertaken is both cognitive and social-emotional activities. To understand the role of the Messenger in shaping our collaborative partnerships and meeting our targets, our critical reflection accessed our internal processes. In this paper, we are the subjects engaged in collaborative research to write academic papers for publication and secure research and development funding. Through our reflection and using the activity theory, we have identified different factors, particularly the affordances of the Messenger in facilitating our collaborative research work, and how these factors influence our socially and culturally mediated activities to achieve our pre-identified outcomes.

### 3.2. Data collection

This research places our experiences at the centre of data collection (Cohen et al., 2012) through narrative and reflective pieces and the primacy object for analysis. We first developed guides for our reflections. These guide questions draw the following:

1. Our motivation for writing journal articles and applying for research and development grants.
2. Useful features of Messenger that make it better compared to traditional email.
3. Features of Messenger that are inappropriate for collaboration.
4. Additional features of Messenger that can make collaboration better.
5. Factors that increase engagement in Messenger for research collaboration.
6. How Messenger facilitates negotiation in research collaboration.
7. Recommended guidelines for using Messenger for research collaboration.

### 3.3. Ensuring trustworthiness of our research

Trustworthiness is a critical consideration in qualitative research with credibility, transferability, dependability and confirmability (Lincoln and Guba, 1985). We used these four criteria to ensure that our autoethnographic study, although based on our personal experience, has high validity and reliability. We adhered to these criteria throughout the completion of this paper, from reflecting to writing this paper.

We ensured credibility through our prolonged engagement in reflection. We compared our reflections and discussed our competing views. We did not aim to reach a consensus but to acknowledge opposing views and frame them within the broader context of preference for using social media for specific research activity. These processes provide a measure of the truth value of our research, ensuring our findings are correct and accurate. Acknowledging our opposing views and reporting them in the paper enhances our credibility.

In terms of transferability, we used detailed descriptions, showing that our research findings can be applied to other academic collaboration contexts. When reading our responses to our guide for reflection, we negotiated unclear responses that needed a more detailed description. The adequate details of our contexts, purpose, targets, and activities for using Messenger allow our readers to make a judgement about whether our results are applicable to other contexts.

Furthermore, dependability was ensured by providing enough information for other researchers who want to do similar studies. We acknowledge that our views may not have captured the entirety of using Messenger for collaborative research work. Hence, we position this paper as an initial investigation that provides evidence of how it can facilitate deeper academic engagement. Through these processes, we have demonstrated the reliability of our research results.

One potential issue that we encounter is the conformability of autoethnography due to its overemphasis on self-narrative, which compromises its neutrality and the findings are based on our views and experiences. We acknowledged our potential bias and personal motivations in writing the findings. The agreed reflection guides provide data that reflect the information needed to answer our research questions. We did not use any other narratives apart from our individual and negotiated responses to ensure that the data included in this paper reflect our objective view.

### 3.4. Data analysis

We read, coded and sorted our reflections to determine categories that answer our research questions. After four iterations, we grouped our responses based on these codes and selected some quotes to embed in our results section. Our Research Question 1 were answered using two codes—personal and professional reasons. For Research Question 2, these include the features of Messenger that facilitate collaboration, additional features we want to have, and the consequences of using Messenger.

## 4. Results

Our paper aims to provide evidence of how Messenger can be used for specific academic work. Our reflection and analyses of our personal experiences provide critical insights into how Messenger has been pivotal in accomplishing our aims to publish journal articles and secure a research grant. We present our critical reflection below following our guide questions.

### 4.1. Our motivation for research collaboration

This section answers our Research Question 1: *What are our motivations for engaging in collaborative research publication?* Although we differ in our career stages in academia, we share two common reasons for engaging in research collaboration: personal and professional.

#### 4.1.1. Personal reasons

We are both motivated at a personal level to contribute to the knowledge economy. As one of the authors said, “I am happy when my papers get published, and I win grants (CZO).” This is our biggest source of motivation. As one of us narrated:

*I am developing a strong research agenda in curriculum, assessment, evaluation, and teacher education and development. My personal aim is to contribute to discourses on these topics, and develop theoretical and practical knowledge with the ultimate aim of influencing learning and teaching practices of school leaders and teachers (DA).*

In addition, as we are both academics and teach in the area of teacher education and development, “we want to address issues that we have identified in the classroom (CZO),” and “our engagement in research will give me personal satisfaction that I was able to use my research skills to find possible solutions (DA).”

#### 4.1.2. Professional reasons

As we are both employed in academia and are expected to engage in research activities, we “need to meet our performance indicators as part of accountability in our job (DA).” There is also a compelling reason for one of us to future employment:

*As my goal is to work as an academic at one of the prestigious universities, I need to boost my curriculum vitae. I believe that publishing many papers and receiving funding will enhance my research track record. As an early career researcher, I need to build an exemplary research track to secure a better job and eventually for promotions (CZO).*

These personal and professional reasons drive us to find a platform that can enhance our collaboration for writing and publishing more papers. Previously, we have tried to use emails for correspondence, sharing resources, and sending reminders, but “I felt emails lack the human connection. It’s very formal, and there is this feeling that conventions in sending emails need to be observed (DA).” On the contrary, “email is fine, and it stores all our previous messages, easy to search previous messages (CZO).”

### 4.2. The role of messenger in our collaborative research work

This section presents our answer to Research Question 2: *How does Messenger facilitate accomplishing our goals of publishing papers and securing a research and development grant?* Our preference for using Messenger to facilitate our research collaboration is influenced by its affordances. The features of Messenger support our interactions across the stages of writing to publishing research papers.

#### 4.2.1. Navigation

Using Messenger for collaboration takes away the formality of traditional email correspondence. “It is easy to navigate, and I can send a message even anytime I have a question (CZO).” As it is a stand-alone app available on mobile phones, it is easy to use without going to the Facebook app before accessing it. Its capacity “to store messages makes it ideal for shifting from mobile app to computer screen (DA).” This functionality is helpful when you are writing a paper and want to clarify something with your collaborators. There is no need to access the Messenger app on your phone while on your laptop or computer screen.

The simple layout of Messenger provides easy navigation. The search bar is helpful and makes it “easy to search someone or a group of people in the Messenger app and shoot a message anytime, anywhere (DA).” On the upper right-hand side features a phone icon that makes it easy to ring someone if a call is needed for further discussion.

##### 4.2.1.1. Convenience

It is a very convenient platform for sharing files, links, photos, and screenshots. We both agree that the drag-and-drop function of

*If you are logged in to your computer, the links, files, and screenshots shared are easy to view without changing a window. You can read a certain section of the file shared and copy and paste in Messenger to facilitate thorough discussion or to support your viewpoint when a certain issue on the paper is being debated upon. It is also easy to drop a screenshot or take a photo when your argument needs further support (DA).*

When you make mistakes, it is easy to retrieve the message by deleting it and editing it before resending it. Also, it is easy to remind your collaborator if they miss an important message for discussion, “you can go back to your message and swipe left, then type a gentle nudge (DA).” In addition, the notification functions of Messenger are helpful, “getting your attention that someone sent you a message. It prompts you and gives you the push to check it and then reply (CZO).”

#### 4.2.2. Synchronous and asynchronous chat

Messenger can integrate online and offline engagement. It provides an opportunity for synchronous and asynchronous discussions, resulting in dynamic and more focussed interactions. The synchronous chat simulates real-time conversation that allows “real-time feedback for our draft, facilitating broad, deep, and ongoing interactions (DA).” Also, “the synchronous chat makes it easier to negotiate on some issues in our research and papers and to reach a consensus if we needed to (CZO).” The “asynchronous chat is handy in our context as we are located in different time zones (CZO).” The 4-h difference presents a challenge for synchronous chat, and hence, “we just send messages any time of the day, and we reply at the time convenient for us (DA).” Although the synchronous chat is ideal than asynchronous chat, there is no expectation that you have to reply even when you see the message. The “seen” label is a great feature that signals the sender that the other person has read the message. “If you see that your message is seen, and there is no reply, then it signals that they are busy or in the middle of something (DA).”

#### 4.2.3. Extra features

There are features that traditional email cannot do. These include making phone calls and using emojis and stickers, which enable more socially cohesive interactions. As summarised:

*When typing a message takes longer, and the competing viewpoint needs to be discussed thoroughly, a voice call is just a click away, without worrying about the expensive overseas charges as in the case of the regular phone call (DA).*

We can express our emotions or feelings in the chat using emojis. Easy to let your collaborator know if you are feeling overwhelmed, happy, shocked, or sad about the paper. Easy to react to the message sent as well without typing what you want to say. The emojis “add life to the conversation as it gives emotions to the conversation that rather becomes intense especially when disagreements build up (CZO).” In addition, the emojis function for social cohesion as they are:

*authentic device to express what you think well. Writing research papers is not mechanistic or routinary, but involves lots of emotions and requires social support. The emojis allow you to express your appreciation, frustrations, and many other feelings (DA).*

#### 4.2.4. Accessibility

The accessibility of Messenger on mobile phones further facilitates our interactions:

*Even when I am on public transport, and I think of something critically important for the project we are writing, I can quickly grab my phone and send message to CZO. It also serves as an excellent note-taking tool to dump all your ideas when you are on the go (DA).*

Also, it is easy to switch to your Facebook account to share your research output or work for wider reach and visibility. If our paper gets accepted, we post the link to the article to our Facebook account for wider reach.

The concept of accessibility in this research context can also be used to highlight the ability of Messenger to list the shared media, files, and links in its Html version. As we continuously engage in research and writing papers, we bring more resources to the conversation, including references for academic writing, exemplars of journal articles, methodology papers, and many other helpful resources to progress with our work.

#### 4.2.5. Additional features of messenger for better collaboration

Although Messenger is loaded with features that facilitate our interactions, there are features that we want to see, including the capability to pin important messages for easier retrieval in the future. Also, it would be nice to see a recording capability of Messenger to record calls and discussions for reviewing in the future. It would be nice also if we could send a calendar invite or a reminder for meetings. In addition, it would be good if the messages could be organised based on threads or topics. This would be particularly helpful if multiple projects are discussed.

#### 4.2.6. Cons of using messenger

As Messenger is inherently for social interactions, there is a high tendency for the conversation to go off the academic topic. On several occasions, our conversation had fallen off the topic, and we discussed other stuff unrelated to our research topic. There is also the risk of deleting the conversation, and once deleted, the entire thread is deleted. There is no restore button to retrieve the deleted thread. Also, some of our collaborators have no Facebook account. Hence, we have to revert to traditional email correspondence, which makes communication relatively slow, especially since we are from different time zones.

Throughout the period of our collaboration, we have negotiated the following guidelines for a more effective use of Messenger.

##### 4.2.6.1. Expectations

• Everyone needs to check their Messenger regularly.
• If they are on leave or holiday, they need to inform in the chat of the period that they will not access Messenger.
• When asked in the chat, they need to reply to the thread for focussed discussion.
• There is no expectation for an immediate reply. You can reply at your convenient time. It must be recognised that everyone is working full-time with various commitments.

##### 4.2.6.2. Rules for engagement

• Set boundaries. Collaborators must agree that the chat is restricted only to academic discussions. When the chat goes off the aim of the collaboration, then someone should call out and steer the conversation back to research.
• When responding to a message, the reply button should be used to establish a thread for individual messages.
• When starting a new conversation or a new topic, signal that it is unrelated to the previous messages.
• Although an immediate reply is not expected, collaborators should agree for a period to reply to any questions. In our case, we should reply within 48 h. After such a period, a reminder should be sent.
• We have never encountered profanity or inappropriate language use in our interactions. However, it must be agreed that inappropriate and offensive language use, bashing, and disrespectful behaviour must not be tolerated.
• Do not delete or delete any messages. Swipe it and retype the correct message.

##### 4.2.6.3. Practical action

• Summarise key insights from the discussion. Share the summary with your collaborator and ask them if it captures all the insights.
• For shared files, photographs, and screenshots, download and save those important in your drive. This applies to key points raised at any point in the chat.

## 5. Discussion

Our auto-ethnographic study aims to reflect on our experience to address the paucity in the literature on how Messenger can be better used for collaborative research to increase our publications and apply for a research and development grant. Our research contributed to the growing body of literature on using social media for collaborative research (Thelwall and Kousha, 2015; Kuteeva, 2016; Onuoha et al., 2021).

As shown in our results, we can leverage the well-established functions of social media for professional development (McPherson et al., 2015; Dermentzi et al., 2016), research collaboration (Jordan and Weller, 2018), enhancement of their reputation (Knight and Kaye, 2016), networking (Dermentzi et al., 2016; Donelan, 2016), sharing of research output (Elsayed, 2016) to a more strategic research collaboration by using it as a medium for writing and negotiating journal articles papers.

We interpret our reflections through the lens of activity theory, using its various components to highlight significant findings. Based on our reflection, achieving our goal, which is the *object* of our collaborative research work to write and publish journal articles, and a secure research and development grant requires that we have clearly established our motivation to engage in this work. Both personal and professional motivations (Zhou et al., 2022) are critical to establishing and sustaining our partnership. These sources of motivation provided the impetus for our collaborative research work and for finding an accessible and useful social media platform for our engagement.

The *object* of our collaboration was negotiated with a realistic expectation regarding the number of published papers in 1 year. We target top-quality journals, quartiles 1 or 2 only, to establish a benchmark for the quality of our work. As we are both the *subjects* for this engagement, it was easier to negotiate the goals and reach a consensus. The role of negotiation in research collaboration is critical (Hake and Shah, 2011), and in our engagement, Messenger provided the platform for negotiating all aspects of writing to publishing.

Messenger is the main *tool* for this collaborative research work, which also provides an overarching function for all research engagements. The affordances of Messenger provide the social aspects of collaboration, including initiating discussions, negotiating leadership, functions and contributions. It also provides administrative functions reminding collaborators to comment on issues and questions and complete the assigned tasks. Moreover, it functions as a repository of conversations, ideas, resources and meta-data of the chat. More broadly, it offers the technological component required for sharing resources, including other tools for collaboration like templates, academic writing resources and journal articles. The benefits of these social media functions in enhancing academic collaboration have been reported in the literature (Gorska et al., 2020; Onuoha et al., 2021).

The internal mechanisms constituted by the *rules* like conceptualising relevant research papers only, use of journal templates, targeting top journals, leadership and contribution for each paper, and the rules for engagement in the Messenger emerged through a collective agreement. They are agreed upon underpinned by our strong belief that they will help us achieve our aim. These rules have created clarity of expectations and built positive relationships between us. These rules develop over time and change upon negotiation to facilitate better collaboration. The rules followed by researchers in using social media for research collaboration enforce ethical norms and advance the professional practice (Zimba et al., 2020).

The partnership we have created built the *community* we need to achieve our goal. We have networked with the broader academic community to seek advice and feedback from other experts in our field. We have created another group chat in Messenger with these experts but with less expectations from them to engage. For others who prefer to use their email, we send our enquires or draft for their feedback. Our engagement with other experts is seen to be valuable as both of us are early career researchers, and we recognise that we need support through mentoring. Expanding our community to support our work is seen as critical in any collaboration’s success (Jordan, 2022).

**FIGURE 1:**
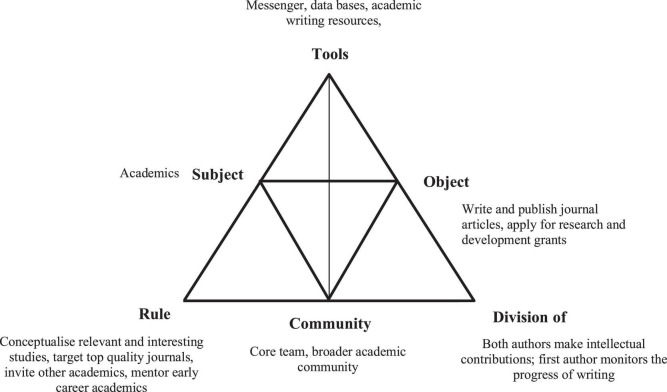
The interrelationships of the factors that contribute to the success of collaborative journal article writing.

Our intellectual contributions, leadership for completing, and administrative functions from conceptualising to submitting our paper are negotiated and clearly articulated as *the division of labour*. Our specific roles for each paper constitute our professional contribution to our collaborative research work. The clarity of the division of labour and the trust we have established have enhanced our responsibility in our engagement. The clear articulation of roles builds partnership and avoids conflicts (Bagshaw et al., 2007), while the trust built among collaborators addresses power imbalances (Kerasidou, 2019). The relationships of these factors are illustrated in Figure 1.

As shown in Figure 1, the research collaboration we formed to build our publication and other research engagement is mediated by the rules, tools, division of labour, and the community we have established. The division of labour and the rules are negotiated and have emerged as a strategy to meet the object or goal of the activity. Negotiation is an ongoing process; thus, the division of labour and rules shift over time. This is an important feature of collaboration as the leadership of authorship in papers changes based on our interest and capability to lead. Over time, we have identified tools apart from Messenger. The tools we have identified and used include conceptual tools (e.g., academic writing resources, methodology papers), material tools (e.g., templates, exemplars of journal articles), and cultural tools (e.g., guidelines for using Messenger). The division of labour, rules, tools, and community shape how we, the subject, orient our collaboration to achieve our own goals. The activity systems we have created is a safe place for interaction as we constantly engage in negotiations to address issues and negotiate tensions among different elements of the systems. From the activity theory, meeting our goals involve resolving contradictions among elements, allowing ourselves to take advantage of an expanded range of actions, including access to a broader repertoire of mediational means, taking turns as lead authors, and developing more efficient ways to work with existing constraints.

A critical aspect of our experience is the rule of Messenger. The activity systems we have created operate within the platform provided by the Messenger app. This is an important contribution of our paper as it highlights the interactions between social media messaging app and activity systems aimed at enhancing our research track record by writing and publishing journal articles and applying for research and development grants. The Messenger serves as the platform for establishing our identity as research collaborators. In our papers, we switch roles in authorship, and whoever is the lead author manages the team.

Overall, using the activity theory to analyse our reflection highlights specific requirements for using Messenger as a collaborative research tool for writing and publishing journal articles. First is the emotional factor particularly motivation and aspiration. This factor is critically important for achieving goals (Osterloh and Frey, 2000). Second, the social factor, including between collaborators and the wider academic network, provides support not only in terms of encouragement but also in providing expert knowledge and critical insights. Third, the personal factor, particularly knowledge and skills, including understanding of the aim of collaboration, specific research knowledge and skills, and leadership and administrative capabilities, provides the expertise required for research activities. Fourth, the structural factor, referring to the tools and rules that facilitate the attainment of our goal, makes the collaboration more cohesive with negotiated expectations. Fifth, the technological factor, using Messenger extensively as a collaborative tool for discussion and negotiation, a repository of recourses and an accessible administrative tool, enhances collaboration and negotiation. Lastly, organisational factor is critical for the success of any collaborative work (Valaitis et al., 2018). Our institutions provided access to databases, working space, and computer programs for data analysis, referencing, and plagiarism checks.

## 6. Conclusion

Our paper has demonstrated how a specific social media platform can be used for more focussed research collaboration to increase our publication and secure a research and development grant. We used the activity theory to highlight the interrelationships of factors (i.e., personal, social-emotional, structural, technological, and organisational) contributing to the success of our engagement. The affordances of the Messenger facilitated our collaborative research work by influencing our socially and culturally mediated activities to achieve our pre-identified outcomes.

As we have adopted an auto-ethnographic research design, which is limited only to our collective reflection, research involving a more sophisticated research design is needed to provide more evidence of the intersections of social media and collaborative research work. Also, we used only one specific social media platform. It is worthwhile to explore other social media that might have been used by other academics but are not reported in the literature.

Although our study has limitations in terms of methodology, we have provided an illustrative overview of the role of Messenger in research collaboration. We have demonstrated how this social media platform can be a valuable tool to help support academics engage in writing and publishing journal articles.

## Data availability statement

The raw data supporting the conclusions of this article will be made available by the authors, without undue reservation.

## Ethics statement

Ethical review and approval was not required for the study on human participants in accordance with the local legislation and institutional requirements. Written informed consent for participation was not required for this study in accordance with the national legislation and the institutional requirements.

## Author contributions

DA: conceptualisation, methodology, data collection, data analysis, and writing – reviewing and editing. CO: conceptualisation, data collection, data analysis, and writing – reviewing. Both authors contributed to the article and approved the submitted version.
